# Effect of resistance exercise on serum leptin levels in a prospective longitudinal study of women patients with rheumatoid arthritis

**DOI:** 10.1186/s13075-022-02765-2

**Published:** 2022-03-26

**Authors:** Young Bin Joo, Kyoung Bo Lee, Bomi Sul, Hye-Soon Lee, Seong Hoon Lim, Yune-Jung Park

**Affiliations:** 1 Division of Rheumatology, Department of Internal Medicine, Hanyang University Guri Hospital, Guri, Republic of Korea; 2 Department of Rehabilitation Medicine, St. Vincent’s Hospital, College of Medicine, The Catholic University of Korea, Seoul, Republic of Korea; 3 Hanyang University Institute for Rheumatology Research, Seoul, Republic of Korea; 4 Division of Rheumatology, Department of Internal Medicine, St. Vincent’s Hospital, College of Medicine, The Catholic University of Korea, Seoul, Republic of Korea

**Keywords:** Leptin, Exercise, Rheumatoid arthritis

## Abstract

**Background:**

Exercise has an anti-inflammatory effect and reduces fat mass. Leptin has been known to be proinflammatory adipokines mainly produced by adipocytes. However, few studies have investigated the association between exercise and changes in serum leptin levels of patients with RA. This study evaluated the effect of an individualized resistance exercise on inflammatory markers including leptin as well as muscle strength and exercise capacity in patients with rheumatoid arthritis (RA).

**Methods:**

A total of 42 age- and sex-matched participants were assigned to a resistance exercise program (60 min, once a week for 12 weeks, and self-exercise twice a week) or to a control group. Muscle strength, exercise capacities, and inflammatory markers such as cytokines and adipokines were assessed at baseline and at 12 weeks follow-up. Longitudinal changes in muscle strength, exercise capacity, cytokines, and adipokines between groups were tested with repeated measures analysis of variance or using the generalized estimating equation, with adjustment for baseline disease activity score 28-C response protein as a covariate.

**Results:**

A total of 37 of 42 female patients with RA completed this prospective intervention study. Grip strength improved significantly in the exercise group (*P* < 0.05), while no between-group changes were found. Quadriceps contraction power (*P* for group-time interaction = 0.035 for the right side and *P* for group-time interaction = 0.012 for the left side) and 6-minute walking distance (*P* for group-time interaction = 0.021) were all improved significantly in the exercise group compared with the control group. In addition, serum leptin levels were significantly decreased in the exercise group compared with the control group (*P* for group-time interaction = 5.22 × 10^−5^), but not the other cytokines or adipokines. The change in serum leptin levels correlated with the changes in fat mass (Rho = 0.491, *P*= 0.015) and visceral fat area (Rho = 0.501, *P*= 0.013).

**Conclusion:**

In addition to muscle strength and exercise capacity, the 12 weeks of individualized resistance exercise reduced serum leptin levels in keeping with body fat mass or visceral fat area, suggesting that serum leptin levels might be a surrogate marker of exercise in RA.

## Introduction

Rheumatoid arthritis (RA) is a chronic inflammatory disease in which articular and extra-articular tissue can be damaged. Uncontrolled inflammatory burden in RA results in loss of muscle mass, known as rheumatoid cachexia [1, 2]. Physical inactivity due to pain or deformity also accelerates muscle atrophy and muscle strength. Control of systemic inflammation with antirheumatic drugs is key for the management of RA but also non-pharmacologic management such as exercise increases the beneficial effects of antirheumatic drugs.

Exercise has numerous benefits in RA. Based on previous reports, exercise improves disease-related outcomes such as fatigue,[3] functional disability [4], and disease activity [4, 5], but also reduces the risk of cardiovascular disease [6–8]. Exercise also improves body composition or muscle strength and ameliorates the complications of rheumatoid cachexia. Especially, exercise is known to have anti-inflammatory effects on chronic inflammatory diseases such as RA [9, 10]. However, few studies have elucidated the mechanism by which exercise improves inflammation in RA or the association between exercise and inflammatory markers.

It has been reported that serum leptin levels, which are considered as proinflammatory adipokines, increased in patients with RA compared with healthy controls although the results showed some discrepancies [11]. Several authors have reported that leptin levels correlated with disease activity measured by disease activity score 28 (DAS28) [12]. In addition, serum leptin levels were associated with an aggressive course of RA in other studies [13–15]. In a recent meta-analysis reported by Lee et al., leptin level was increased in RA compared with healthy controls and correlated with disease activity, suggesting that serum leptin might play a role in RA pathogenesis. However, limited data were available to investigate the association between exercise and the change in serum leptin levels in RA, although exercise appears to have anti-inflammatory effects and reduced fat mass, which is the primary origin of leptin production.

We hypothesized that a resistance exercise reduces serum leptin levels in patients with RA. The individualized resistance exercise program was conducted in patients with RA for 12 weeks and the changes in serum leptin as well as other inflammatory cytokines were compared with control groups to identify the effects of exercise in RA.

## Methods

This prospective intervention study was conducted between Aug 2017 and Aug 2018. This study was approved by the Institutional Ethics Review Board (IRB) of St. Vincent’s Hospital, Catholic University of Korea (VC18FESI0049). All patients provided written informed consent approved by the participating institutions. The study protocol was registered with Clinical Research Information Service (KCT0004110), where aims of the study protocol are to investigate the association of a resistance exercise with body composition in patients with RA while also designing a study to evaluate the association of changes in the inflammatory status with a resistance exercise. Among those, this study aimed to show the association of changes in the inflammatory status with a resistance exercise.

### Participants

Patients with RA aged between 18 and 65 years were selected as the study subjects. Only women with RA were enrolled considering the differences in body composition by sex. Patients with RA fulfilling the 2010 American College of Rheumatology (ACR) classification criteria [16] who have not been actively exercising within the last 3 months were on a stable dose of disease-modifying antirheumatic drugs (DMARDs) and corticosteroids for at least 3 months prior to screening, and ACR functional class [17] I to III were enrolled. Patients were excluded if they were diagnosed with new chronic diseases such as diabetes mellitus or cancers within the last 3 months, diagnosed with unstable angina or myocardial infarction within the past 1 month, or had difficulty with rehabilitation exercise program due to unstable cardiovascular disease or severe disease, in particular, systolic blood pressure more than 180 mmHg or diastolic blood pressure more than 100 mmHg, or heart rate more than 120 at rest, underwent hip or knee joint replacement, and ineligible for rehabilitation exercise based on medical judgment.

### Interventions

Eligible individuals were assigned to either a resistance exercise group or a control group. The patients in the exercise group participated in an exercise program for 60 min once a week for 12 weeks and performed self-exercise twice a week. The exercise program included a warm-up stretch exercise for 15 min and a resistance exercise for 45 min. The warm-up stretch exercise included shoulder and deltoid, biceps and wrist flexor, quadriceps, hamstring and low back, groin, calf, upper back, neck flexor, and neck rotator stretch and repeated two times. The resistance exercise using an elastic band included 8 items based on guidelines provided by the American College of Sports Medicine (ACSM) for older adults [18]: resistance band squats, resistance band bent-over rows, alternate chest presses on standing, diagonal woodchops, triceps extension with resistance band, resistance band lunges, lateral rows with resistance band, and biceps curls with resistance band. Each item of resistance exercise was repeated 15 times per set and performed in three sets with a rest of 2 min between exercise sets. Exercise intensity was gradually increased using different elastic bands ranging from yellow (the lowest resistance) to red and green colors (TheraBand, Akron, USA). During the first 4 weeks, the TheraBand with the lowest resistance was used to perform muscle resistance exercises, followed by medium resistance TheraBand by week 5. If it is difficult to perform more than 10 times per set, the previous resistance level of TheraBand was continued. Exercise programs were supervised by a professional exercise physiologist.

The control group was asked to abstain from new external exercise programs. However, this group participated in preexisting recreational activities.

### Clinical factors

Clinical factors assessed in both groups included age, height, weight, RA-related characteristics including disease duration, the presence of rheumatoid factor and anti-cyclic citrullinated protein (anti-CCP) antibody, disease activity score 28-erythrocyte sedimentation rate (DAS28-ESR), DAS28-C reactive protein (CRP), and medications such as conventional DMARDs and biologics. DAS28-ESR and DAS28-CRP were measured before and after exercise in the exercise group, at the time of enrollment, and 3 months later.

### Body structure and function assessment

Changes in muscle strength were measured before and after exercise in order to determine whether resistance exercise for 12 weeks helped patients to improve their strength. A 6-min walking test (6MWT, unit = meters) was also performed to confirm the effect of exercise on patients with RA. Upper extremity muscle strength was measured by evaluating the grip strength of both hands using an electronic hand grip meter (model No. KH-100, KYUNG IN, Korea, unit = lbs.) and the mean grip strength based on three measurements was recorded. Lower extremity muscle strength was measured via isometric quadriceps contraction using a handheld dynamometer (JM-CM305, Jtech Medical, USA, unit = lbs.). A maximal isometric quadriceps contracture in a seated upright position with the knee placed in 90° flexion was performed via three attempts on each side and the mean value was recorded. A trained and experienced therapist (LKB) assessed all the muscle strength measurements as well as conducted the exercise sessions. The 6MWT measures the maximum distance walked over 6 minutes, which was performed twice with sufficient rest, and the best performance was selected as a record.

### Inflammatory cytokines and adipokines

Fasting blood samples were collected in the morning from both exercise and control groups. Samples were centrifuged at 14,000×*g* for 10 min at 4°C immediately after collection, and the clarified supernatants were aliquoted into sterile 1 mL tubes and stored at − 70 °C until use. The concentrations of interleukin (IL)-1, IL-6, tumor necrosis factor (TNF)-a, leptin, and adiponectin in supernatants were measured using a commercial enzyme-linked immunosorbent assay (ELISA) kit (R&D Systems), according to the manufacturer’s recommendations.

### Body composition assessment

To investigate the association between changes in serum inflammatory cytokines and changes in body composition, we measured the body composition before and after 12 weeks of exercise in women with RA via direct segmental measurement bioelectrical impedance analysis (an eight-polar tactile-electrode impedance meter, nBody S10, Seoul, Korea). For each patient, baseline and follow-up scans were obtained using the same instrument.

### Statistical analysis

Baseline characteristics were compared using Wilcoxon signed-rank test or Fisher’s exact test. Longitudinal changes in muscle strength and 6MWT between groups were tested via repeated measures analysis of variance, with adjustment for baseline DAS28-CRP as a covariate. Longitudinal changes in cytokines and adipokines between groups were tested using the generalized equation, with adjustment for baseline DAS28-CRP as a covariate. All tests were two-sided and *p* values less than 0.05 were considered to indicate statistical significance. All statistical analyses were performed using PASW Statistics, version 17 (PASW, Chicago, IL, USA).

## Results

### Baseline demographic characteristics

**Fig. 1:**
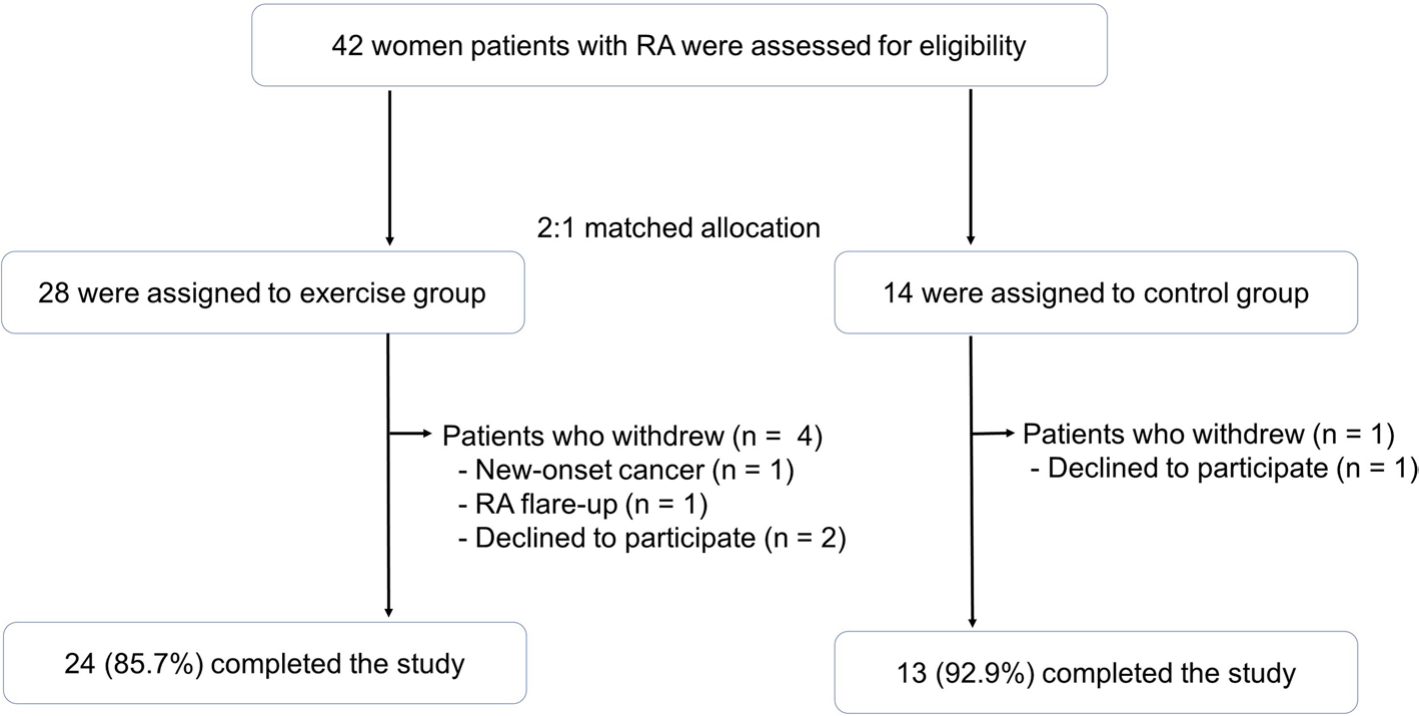
Study flow chart. RA, rheumatoid arthritis

A total of 42 participants were enrolled, and 37 (88%) of them completed the study (Fig. 1). In the exercise group, the mean age ± standard deviation (SD) was 51.2 ± 6.9 years with disease duration of 6.7 ± 7.5 years (Table 1). BMI was 24.7 ± 3.7 kg/m^2^ and fat mass, fat-free mass, and the visceral fat area was 22.9 ± 7.4 kg, 38.3 ± 3.4 kg, and 117.2 ± 43.5 cm^2^, respectively. C-reactive protein (CRP) was 0.4 ± 0.8 mg/dl and DAS28-CRP was 2.5 ± 0.7. As a RA medication, 29.2% has been treated with conventional DMARDs (cDMARDs) monotherapy and 50% with cDMARDs combination therapy. Biologics was used in 20.8% of patients. In the control group, the mean age was 47.6 ± 9.3 years with a disease duration of 4.4 ± 7.4 years. BMI was 23.9 ± 4.3 kg/m^2^, and fat mass, fat-free mass, and the visceral fat area were 21.8 ± 8.1 kg, 40.8 ± 5.8 kg, and 106.3 ± 46.2 cm^2^, respectively. C-reactive protein (CRP) was 0.1 ± 0.1 mg/dl and DAS28-CRP was 2.1 ± 0.9. As a RA medication, 23.1% has been treated with cDMARDs monotherapy and 69.2% with cDMARDs combination therapy. Biologics was used in 7.7% of patients. Among the baseline factors, DAS28-CRP of the exercise group was significantly higher than that of the control group (*P* = 0.047). Excluding DAS28-CRP, factors at baseline were not significantly different between exercise and control groups.

**Table 1:** Comparison of exercise and control group at baseline

	Total (*n* = 37)	Exercise group (*n* = 24)	Control group (*n* = 13)	*P*
Demographic				
Age, years	49.9 ± 7.9	51.2 ± 6.9	47.6 ± 9.3	0.258
Anthropometric				
Height, cm	158.4 ± 4.9	157.5 ± 4.6	159.9 ± 5.3	0.122
Weight, kg	61.4 ± 10.5	61.4 ± 8.8	61.5 ± 13.5	0.310
Body mass index, kg/m^2^	24.4 ± 3.8	24.7 ± 3.7	23.9 ± 4.3	0.417
RA-related characteristics				
Disease duration, years	5.9 ± 7.4	6.7 ± 7.5	4.4 ± 7.4	0.070
Rheumatoid factor positivity	28 (75.7)	19 (79.2)	9 (69.2)	0.423
Anti-CCP antibody positivity	30 (81.1)	21 (87.5)	9 (69.2)	0.423
ESR, mm/h	20.7 ± 15.2	22.5 ± 14.4	17.3 ± 16.7	0.325
CRP, mg/dl	0.3 ± 0.7	0.4 ± 0.8	0.1 ± 0.1	0.226
DAS28-ESR	3.0 ± 1.0	3.2 ± 0.8	2.6 ± 1.2	0.070
DAS28-CRP	2.4 ± 0.8	2.5 ± 0.7	2.1 ± 0.9	0.047
RA medication				0.413
cDMARD monotherapy	10 (27.0)	7 (29.2)	3 (23.1)	
cDMARD combination	21 (56.8)	12 (50.0)	9 (69.2)	
Biologics	6 (16.2)	5 (20.8)	1 (7.7)	
Body composition				
Fat mass, kg	22.5 ± 7.6	22.9 ± 7.4	21.8 ± 8.1	0.683
Fat free mass, kg	39.2 ± 4.5	38.3 ± 3.4	40.8 ± 5.8	0.115
Visceral fat area, cm^2^	113.3 ± 44.2	117.2 ± 43.5	106.3 ± 46.2	0.482

Values are presented as *N* (%) or mean ± standard deviation.

*P* values are for the difference between groups as calculated with the use of the Wilcoxon signed-rank test or Fisher’s exact test.

*Abbreviations*: *RA* rheumatoid arthritis, *anti-CCP* anti-cyclic citrullinated peptide, *DAS28-ESR* disease activity index 28-erythrocyte sedimentation rate, *DAS28-CRP* disease activity index 28-C-reactive protein, *cDMARD* conventional disease-modifying antirheumatic drug

### Changes in muscle strength and exercise capacity after resistance exercise

To ensure that resistance exercise was performed properly, we first assessed the changes in muscle strength and exercise capacity after 3 months of exercise in both groups. After 3 months of resistance exercise, muscle strength in upper extremities measured via grip strength improved significantly in the exercise group (Table 2). Grip strength of the right hand significantly increased from 43.8 ± 2.4 lbs to 46.6 ± 2.2 lbs (*P* < 0.05), and that of the left hand also increased from 44.3 ± 2.3 to 47.3 ± 1.7 (*P* < 0.01) in the exercise group. However, no changes in grip strength were observed in the control group. In the control group, grip strength of the right hand was 55.7 ± 3.2 lbs in baseline and 54.0 ± 3.0 lbs after 3 months (*P* > 0.05), and that of the left hand was 50.2 ± 3.1 and 48.0 ± 2.3 after 3 months (*P* > 0.05).

**Table 2:** Changes in muscle strength and exercise capacity from baseline to 3-month time-point for exercise versus control groups

		Exercise group	Control group	F	*P* (group-time interaction)
Upper extremity	Grip strength, Rt				
	Baseline, lbs	43.8 ± 2.4	55.7 ± 3.2		
	Change at 3 months, lbs	46.6 ± 2.2*	54.0 ± 3.0	2.046	0.119
	Grip strength, Lt				
	Baseline, lbs	44.3 ± 2.3	50.2 ± 3.1		
	Change at 3 months, lbs	47.3 ± 1.7**	48.0 ± 2.3	3.117	0.086
Lower extremity	Muscle strength, Rt				
	Baseline, lbs	53.6 ± 1.5	56.8 ± 2.0		
	Change at 3 months, lbs	60.7 ± 1.4**	58.1 ± 2.0	4.815	0.035
	Muscle strength, Lt				
	Baseline, lbs	53.0 ± 1.3	54.6 ± 1.8		
	Change at 3 months, lbs	58.7 ± 1.5**	55.0 ± 2.0	7.026	0.012
Exercise capacity	6MWT				
	Baseline, m	495.9 ± 9.9	508.4 ± 13.5		
	Change at 3 months, m	528.8 ± 11.4**	523.2 ± 15.4*	5.866	0.021

Values are presented as estimated means ± standard error from the repeated-measures analyses of variance

*P* values are for the interaction between groups over time (from baseline to 3 months) as calculated with the use of repeated-measures analyses of variance (with baseline DAS28-CRP as a covariate)

*Abbreviations*: *6MWT* 6-min walking test

*Significant difference to baseline *p*<0.05 (repeated measures ANOVA)

**Significant difference to baseline *p*<0.01 (repeated measures ANOVA)

Lower extremity muscle strength measured via isometric quadriceps contraction was also improved in the exercise group, in which muscle strength of the right quadriceps significantly increased from 53.6 ± 1.5 lbs to 60.7 ± 1.4 lbs (*P* < 0.01), and that of the left quadriceps significantly increased from 53.0 ± 1.3 lbs to 58.7 ± 1.5 lbs. In the control group, however, muscle strength of the right quadriceps was 56.8 ± 2.0 in baseline and 58.1 ± 2.0 (*P* > 0.05) after 3 months and that of the left quadriceps was 54.6 ± 1.8 in baseline and 55.0 ± 2.0 after 3 months (*P* > 0.05).

When comparing the difference in change of muscle strength between the exercise and control group, there was no difference in the change in upper extremity muscle strength (*P for group-time interaction* = 0.119 for right upper extremity and *P for group-time interaction* = 0.086 for left upper extremity), but the change in lower extremity muscle strength was significantly higher in the exercise group than in control group (*P for group-time interaction* = 0.035 for right lower extremity and *P for group-time interaction* = 0.012 for left lower extremity).

The change in a 6MWT distance, which represents exercise capacity, was increased after 3 months in both groups. The 6MWT distance increased from 495.9 ± 9.9 m to 528.8 ± 11.4 mg in exercise group (*P* < 0.01) and from 508.4 ± 13.5 to 523.2 ± 15.4 in control group (*P* < 0.05). However, the change of a 6MWT was significantly higher in the exercise group than in the control group (*P for group-time interaction* = 0.021).

### Effect of resistance exercise on changes in inflammatory cytokines and adipokines

First, accurate performance of resistance exercise was established via assessment of improvement in both muscle strength and exercise capacity. Next, the primary outcome of the effect of resistance exercise in decreasing the inflammatory burden on patients with RA was evaluated. Serum levels of IL-1, IL-6, and TNF alpha showed no changes after resistance exercise (*P* > 0.05, Table 3). Serum leptin decreased from 174.3 ± 25.8 ng/mL to 119.3 ± 15.0 ng/mL (*P* = 0.008) after 3 months of exercise. However, the level of serum leptin showed no significant changes (123.6 ± 20.0 ng/mL to 124.8 ± 24.7 ng/mL) in the control group (*P* > 0.05). The difference in the levels of serum leptin between exercise and control groups was significant (*P for group-time interaction* = 5.22 × 10^−5^).

**Table 3:** Effect of a resistance exercise on inflammatory cytokines and adipokines

	Exercise group	Control group	Wald *Χ*^2^	*P* (group-time interaction)
IL-1, pg/ml				
Baseline	1.7± 0.1	7.3 ± 3.0		
Change at 3 months	1.8 ± 0.2	6.7 ± 2.4	2.095	0.148
IL-6, pg/ml				
Baseline	3.3 ± 0.5	5.6 ± 2.8		
Change at 3 months	4.5 ± 1.1	7.7 ± 3.3	0.044	0.835
TNF alpha, pg/ml				
Baseline	3.6 ± 0.5	2.8 ± 0.2	0.020	0.886
Change at 3 months	3.7 ± 0.8	2.9 ± 0.2		
Leptin, ng/ml				
Baseline	174.3 ± 25.8	123.6 ± 20.0		
Change at 3 months	119.3 ± 15.0*	124.8 ± 24.7	16.367	5.22×10^−5^
Adiponectin, ug/ml				
Baseline	1.8 ± 0.3	1.4 ± 0.3		
Change at 3 months	1.9 ± 0.4	1.7 ± 0.2	0.303	0.531

Values are presented as means ±SE

*P* values are for the interaction between groups over time (from baseline to 3 months) as calculated with the use of the generalized estimating equation (with baseline DAS28-CRP as a covariate)

*Abbreviations*: *IL* interleukin, *TNF* tumor necrosis factor

*Significant difference to baseline *p*<0.05 (repeated measures ANOVA)

**Fig. 2:**
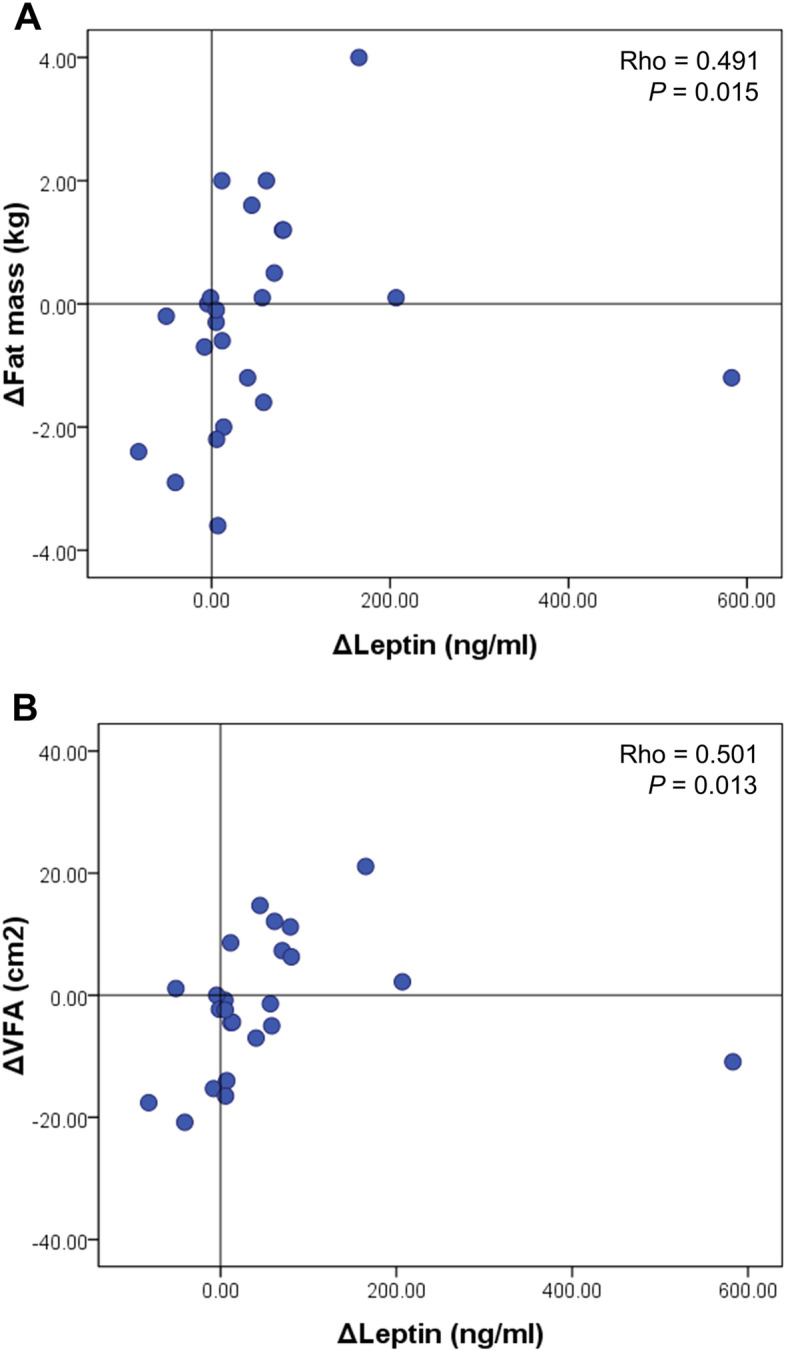
The associations between the change of serum leptin level and the change of fat mass (**A**) and the change of visceral fat area (**B**) after 12-week individualized resistance exercise. VFA, visceral fat area

We further investigated the association between changes in serum leptin and body composition. In the exercise group, the change in serum leptin level was correlated with the change in fat mass (Rho = 0.491, *P* = 0.015, Fig. 2A). The change in serum leptin level was also correlated with the change in the visceral fat area (Rho = 0.501, *P* = 0.013, Fig. 2B).

## Discussion

In this prospective intervention study, we found that resistance exercise using TheraBand for 3 months in patients with RA increased lower muscle strength as well as exercise capacity based on 6MWT compared with the control group. In addition, serum leptin levels decreased significantly after 3 months of resistance exercise in patients with RA. The change in serum leptin level correlated with changes in body fat mass and visceral fat area. To our knowledge, this is the first study demonstrating the effect of resistance exercise on serum leptin levels in patients with RA, using a prospective study design.

The benefits of exercise in patients with RA are obvious. Muscle strength, which is weakened by systemic inflammation, physical inactivity, and adverse effects of drug therapy can be recovered, resulting in a better quality of life. In addition, exercise decreases the risk of cardiovascular disease [6–8], which is important as the main cause of death in patients with RA is cardiovascular disease. However, the guidelines for non-pharmacologic management of RA do not recommend the appropriate dose, frequency, intensity, or time of exercise.

This study conducted an individualized resistance exercise program using TheraBand based on guidelines recommended by the ACSM for older adults, which demonstrated appropriate effects of exercise on muscle strength and exercise capacity. In the study, grip strength representing upper extremity muscle strength improved significantly in the exercise group, while no between-group changes were found. In RA, grip strength can be used not only as a marker of function and disability but also as an outcome measurement in clinical trials. For example, grip strength was improved during the first year of anti-TNF treatment in RA [19]. In addition, lower grip strength was associated with worse prognosis or long-term outcomes in RA [20, 21]. Lower extremity, assessed in terms of quadriceps strength, is associated with bone quality and prevention of bone loss [22], and also improved after exercise. A resistance exercise program used in this study showed obvious improvement in muscle strength and exercise capacity of patients with RA without any adverse effect during 12 weeks of the exercise program. Resistance exercise using TheraBand, and not a gym-based exercise, is convenient and safe to perform at home individually by patients with RA. The type, frequency, and intensity of the exercise conducted in this study offer practical help for patients with RA in the real world, although studies with a larger sample size and a longer disease duration or follow-up are needed to validate the results.

It has been known that exercise lowers serum leptin levels [23–25]. Some studies investigated the change in leptin levels after exercise in other diseases, but not in RA. It remains unclear whether serum leptin levels could be lowered with exercise in patients with RA as serum leptin levels were increased in patients with RA due to chronic inflammatory conditions. This is the first study, to our knowledge, to show that exercise decreased serum leptin levels in RA. In one study involving patients with fibromyalgia, the leptin level was significantly decreased after resistance exercise for 15 weeks in lean women, but not in overweight and obese individuals [26]. The mean BMI in women patients with RA in our study was 24.4, representing overweight which is defined based on criteria for the Asian population. The beneficial effect of exercise on serum leptin in patients with RA is similar to the effect observed in overweight patients with RA, unlike fibromyalgia.

The effects of serum leptin on joint tissue were investigated in an animal model of RA. In leptin-deficient antigen-induced arthritis mice, joint inflammation was decreased and levels of inflammatory cytokines such as IL-1β and TNF were decreased in the synovium of the knee [27]. In another study, leptin injection into mice with collagen-induced arthritis exacerbated joint inflammation and resulted in joint damage [28]. In addition to the proinflammatory effect, leptin mediates both innate and adaptive immunity [11, 29]. In innate immunity, leptin promotes cytotoxicity of natural killer cells; induces activation of granulocytes, macrophages, and dendritic cells; and activates phagocytosis [11, 30]. In adaptive immunity, leptin polarizes T helper cell subsets towards a proinflammatory state and decreases regulatory T cell proliferation [11, 31]. The evidence suggests that control of the leptin signaling pathway plays an important role in modulating disease activity in RA.

The strengths of our study include a well-designed exercise program based on ACSM guidelines for older adults. This exercise protocol suggests appropriate exercise frequency, time as well as type in patients with RA. Also, a professional exercise physiologist (KBL) supervised and educated patients regarding all courses of exercise. In addition, the drop-out rate was low in both exercise and control groups.

Our study has limitations. The lack of a randomized study design is a major limitation. In addition, physical function was assessed by the same person leading the exercise program, which may bias the assessment of the effect of resistance exercise on muscle strength. Also, we could not show any association between changes in serum leptin and changes in disease activity, perhaps due to the low disease activity of participants in the study. Mean DAS28-ESR and DAS28-CRP were 3.0 ± 0.2 and 2.4 ± 0.1, respectively. Furthermore, we could not investigate the cardiovascular risk factors. The association between serum leptin levels and atherosclerosis or cardiovascular disease was investigated in another study unrelated to RA. Also, the beneficial effect of exercise on cardiovascular risk in patients with RA has been reported. However, the precise association between exercise and cardiovascular risk requires a long-term prospective study design, beyond 12 weeks. Further studies are need to investigate the long-term effects of exercise in patients with RA.

## Conclusions

In conclusion, resistance exercise using TheraBand not only improved muscle strength and exercise capacity but also significantly reduced serum leptin levels. As the level of serum leptin correlated with the body fat mass, the changes in serum leptin represent a useful marker to monitor the efficacy of exercise in patients with RA. However, it is not clear whether the change in serum leptin level was due to the adipose tissue. Thus, additional studies are needed to identify the tissues or cells contributing to the decrease in serum leptin levels and investigate the mechanisms underlying exercise and decrease in serum leptin levels in patients with RA.

## Data Availability

No data are available.

## Abbreviations

RA: Rheumatoid arthritis
DAS28: Disease activity score 28
IRB: Institutional Ethics Review Board
ACR: American College of Rheumatology
DMARDs: Disease-modifying antirheumatic drugs
ACSM: American College of Sports Medicine
anti-CCP: Anti-cyclic citrullinated protein
CRP: DAS28-C reactive protein
6MWT: 6-min walking test
IL: Interleukin
TNF: Tumor necrosis factor
ELISA: Enzyme-linked immunosorbent assay
SD: Standard deviation
